# 

**Published:** 1913-01-11

**Authors:** 


### Gifts and Bequests.

The Chelsea Hospital for Women has received £10 10s. from the Broderers' Company and £5 5s. from the Salters' Company.

The Royal Free Hospital, Gray's Inn Road, has received £250 from the Prudential Insurance Company towards the cost of the new out-patient department.

The Royal Dental Hospital, Leicester Square, has received £400 from Messrs. F. W. Emery and F. Nicholls, the executors of the late Captain T. B. Heathorn.

The Royal Sea Bathing Hospital for Surgical Tuberculosis, Margate, has received a legacy of £1,000, which the directors have applied at the request of the executor to the endowment of a bed in memory of the late Joseph Barton.

Chevalier Epifano Rodriguez, sherry shipper and commission agent, has, subject to legacies of about £5,000, left the residue of his estate equally to the Royal Hospital for Incurables at Putney, Charing Cross Hospital, the Metropolitan Hospital, the London Hospital, the Royal Ear Hospital, Soho, and four other charities.

Among the latest contributions received for King Edward's Hospital Fund for London are the following annual subscriptions:—Merchant Taylors' Company, £1,000; Mr. Robert Fleming, £500; Messrs. Baring Brothers and Co., Ltd., £250; Lord Revelstoke, £100; Messrs. Thomas Cook and Son, £50; "I. J. H.," £50; and "Anonymous," £25.

### Doctors' Wills.

Dr. William Tusting Cocking, lately Professor of Materia Medica and Therapeutics in the University of Sheffield, has left estate valued at £19,261 gross, and £13,908 net.

Surgeon-Colonel Samuel Butler Mason, for many years medical officer of health at Pontypool, and surgeon to the Pontypool Hospital and to several colliery companies, has left estate valued at £13,078 gross, and £10,522 net.

Dr. Henry Salt, of Malvern, at one time physician to the St. Pancras and Northern Dispensary, and later to the Bournemouth General Dispensary, has left estate of the value of £30,441 gross, and £28,419 net.

### Buildings Contemplated and in Progress.

Airdrie.—The Town Council has considered a scheme for a new hospital and sanatorium, estimated to cost £11,000, and referred the subject to a committee with a view to reducing the cost.

Berwick.—A remodelling of Berwick Workhouse is projected at a cost of £8,900.

Bradford.—The Local Government Board has approved plans for a new workhouse at Daisy Hill, estimated to cost £7,100.

Devon.—The County Council has decided to purchase Hawkmoor Estate, near Bovey Tracey, for a sanatorium site. The purchase price is £9,250.

Exeter.—The Corporation propose to extend the isolation hospital at Whipton. Plans have been prepared by the City Engineer, Mr. T. Moulding, M.I.C.E. Tenders are under consideration.

Fowey.—Mr. C. W. Parkes Lees is architect for a cottage hospital at Fowey, for which tenders are invited.

Holywell.—Messrs. J. H. Davies and Son, of Chester, are architects for a new infirmary at Holywell, North Wales, for the Guardians. Tenders are under consideration.

London.—Thirteen tenders have been received for works at Wandsworth Workhouse receiving wards. The lowest is £5,973, the highest £7,395.

London.—Mr. William Watson, A.R.I.B.A., of 53 Chancery Lane, W.C., is architect for extensive dilapidation works at the St. George's East Guardians' premises, including an installation of electric light; also for a swimming bath and gymnasium at the Guardians' schools, Green Street, Upton Park.

London.—£8,000 has been subscribed towards the cost of a hospital for Jews at Stepney Green. A site has been obtained, and the completed buildings are estimated to cost £22,000.

London.—The Asylums Committee has recommended to the County Council that the erection and equipment of the Maudsley Hospital at Denmark Hill for the study of mental diseases be proceeded with, subject to the approval of plans by the Secretary of State and the voting of expenditure therefor, and that the estimate of £56,000 submitted by the Finance Committee be approved.

Mexico City.—The National Railways of Mexico project the erection of a main hospital building and four subsidiary buildings in the Popolta Federal District.

Port Talbot.—Mr. F. B. Smith is architect for the Port Talbot and District Hospital. Particulars of tender for the foundation contract may be obtained from him at St. Oswald's Chambers, Port Talbot.

### Books Received.

J. and A. Churchill: "Elementary Clinical Pathology," by G. Herschell and R. Weiss, 2nd edition; "Text-Book of Anatomy for Nurses," by E. R. Bundy, M.D.

Duckworth and Co.: "The Praying Girl," by Ceres Cutting.

H. K. Lewis: "Flatulence and Shock," by F. G. Crookshank, M.D., M.R.C.P.; American "Medical Review of Reviews"; "Pathfinders in Medicine," by V. Robinson.

G. Bell and Sons: "Hoblyn's Dictionary of Medical Terms," by J. A. P. Price, M.D.

The Scientific Press, Ltd.: "Isotonic Sea-water Injections," by Sandberg and Tudor.

John Wright and Sons: "Notes on the Treatment of Tuberculosis."

### Appointments.

Dr. H. E. Newman, M.B., B.Ch., has been appointed assistant house physician at the Devonshire Hospital, Buxton, Derbyshire.

The Leyton District Council have selected, upon the recommendation of the Education Committee, Dr. Alfred Ball for the post of assistant schools medical officer. The salary starts at £250, with an additional £8 per annum for travelling expenses.

### SIR W. DUNN'S BEQUESTS TO PAISLEY.

In addition to the bequest of £15,000 by Sir William Dunn to Paisley, the following gifts are announced:—£2,000 to the Incurable Homes at Meikleriggs, and £1,000 each to the Eye Infirmary, the Paisley Convalescent Homes at West Kilbride, and the Kibble Institution.

